# Longitudinal analysis strategies for modelling epigenetic trajectories

**DOI:** 10.1093/ije/dyy012

**Published:** 2018-02-16

**Authors:** James R Staley, Matthew Suderman, Andrew J Simpkin, Tom R Gaunt, Jon Heron, Caroline L Relton, Kate Tilling

**Affiliations:** MRC Integrative Epidemiology Unit, Bristol Medical School, University of Bristol, Bristol, BS8 2BN, UK

**Keywords:** epigenetics, epigenome-wide association study, longitudinal data analysis, DNA methylation, ARIES, ALSPAC

## Abstract

**Background:**

DNA methylation levels are known to vary over time, and modelling these trajectories is crucial for our understanding of the biological relevance of these changes over time. However, due to the computational cost of fitting multilevel models across the epigenome, most trajectory modelling efforts to date have focused on a subset of CpG sites identified through epigenome-wide association studies (EWAS) at individual time-points.

**Methods:**

We propose using linear regression across the repeated measures, estimating cluster-robust standard errors using a sandwich estimator, as a less computationally intensive strategy than multilevel modelling. We compared these two longitudinal approaches, as well as three approaches based on EWAS (associated at baseline, at any time-point and at all time-points), for identifying epigenetic change over time related to an exposure using simulations and by applying them to blood DNA methylation profiles from the Accessible Resource for Integrated Epigenomics Studies (ARIES).

**Results:**

Restricting association testing to EWAS at baseline identified a less complete set of associations than performing EWAS at each time-point or applying the longitudinal modelling approaches to the full dataset. Linear regression models with cluster-robust standard errors identified similar sets of associations with almost identical estimates of effect as the multilevel models, while also being 74 times more efficient. Both longitudinal modelling approaches identified comparable sets of CpG sites in ARIES with an association with prenatal exposure to smoking (>70% agreement).

**Conclusions:**

Linear regression with cluster-robust standard errors is an appropriate and efficient approach for longitudinal analysis of DNA methylation data.

**Key Messages**

- DNA methylation levels vary over time, and studying these patterns will aid the understanding of the biological relevance of these markers.
- Performing an epigenome-wide association study at each repeated measure time-point will identify the CpG sites with the largest longitudinal associations with the exposure.
- Linear regression with cluster-robust standard errors is an efficient alternative to multilevel models for the longitudinal analysis of DNA methylation data.

## Introduction

Epigenome-wide association studies (EWAS) have been used to investigate the associations between DNA methylation and a wide range of phenotypes and diseases (see Supplementary Material for short summary of EWAS, available as Supplementary Data at *IJE* online).^1^^,^^2^ These analyses tend to be cross-sectional, testing for associations between methylation at CpG sites and the phenotype at one point in time. However, DNA methylation levels are known to vary over time^3^ and modelling these trajectories could aid in understanding the biological relevance of epigenetic change over time.^4^ Previous investigations into epigenetic change have, so far, focused on analysing CpGs that are associated with the phenotype at baseline or at later time-points, as opposed to fitting longitudinal trajectories for all available sites.^5^^,^^6^ These cross-sectional (time-point-specific) approaches are practical and will identify the CpGs with the largest effects at baseline and those sites that diverge the most based on the exposure over time. The drawback, however, is that additional CpGs that have a time-varying association with an exposure might be missed due to the misspecification of the model in relation to the question of interest.

Multilevel models are often used in traditional epidemiology to investigate associations between an exposure and repeated measures of an outcome over time, while accounting for clustering within individuals using random effects.^7^ However, these models are computationally expensive when fitting many separate outcomes, as is the case in DNA methylation data using the Infinium HumanMethylation450 BeadChip (485 000 CpGs).^8^ An alternative approach is to fit linear regression models across time-points and account for the non-independence with cluster-robust standard errors.^9^ Although, these models are less flexible than multilevel models,^10^ they will yield comparable population average estimates and inferences, while being computationally more efficient.^11^

Here, we have compared cross-sectional and longitudinal modelling approaches for identifying CpGs that change over time in relation to an exposure. We first performed a simulation study, and then applied these modelling approaches to investigate the effect of prenatal exposure to smoking on offspring DNA methylation change over childhood and adolescence.

## Methods

### Modelling approaches

#### EWAS

The most widely used approach of identifying epigenetic change over time is to perform an EWAS at baseline, and investigate whether these associations persist over time.^5^^,^^6^ A more comprehensive approach is to perform an EWAS at each time-point and fit the trajectories of those CpGs that are associated with the exposure at one or more time-points.^12^ Another possible approach is to model the trajectories of those CpGs that are associated with the exposure at all time-points.

#### Multilevel models

Multilevel models are often used to model trajectories over time between repeated measures of an outcome and an exposure. These models contain random-effect parameters that model the within-and-between-individual variance components.^7^ Assuming a (between-individual) random intercept and slope for the exposure, then the model takes the form:

$$y_{ij}=\;\left(\beta_{00}+\beta_{0\text{1}}z_{j}+u_{0j}\right)\;+\;\left(\beta_{\text{1}0}+\beta_{\text{11}}z_{j}+u_{\text{1}j}\right)x_{ij}+\varepsilon_{ij},$$

where *y_ij_* and *x_ij_* are the repeated measures of the outcome and age/time for the *i*-th measurement for the *j*-th individual and *z_j_* is the exposure of interest. The *u*’s are the random effects for the intercept and slope, and are assumed to be uncorrelated with *ε_ij_* and *u_j_* ∼ *N*(0, Σ_*u*_) (where Σ_*u*_ is an unstructured covariance matrix) and *ε_ij_* ∼ *N*(0, σ_*e*_^2^).

#### Linear regression with cluster-robust standard errors

Standard linear regression provides valid effect estimates ignoring the repeated measures within individuals:

$$y_{ij}=\;\left(\beta_{00}+\beta_{0\text{1}}z_{j}+u_{0j}\right)\;+\;\left(\beta_{\text{1}0}+\beta_{\text{11}}z_{j}+u_{\text{1}j}\right)x_{ij}+\varepsilon_{ij}.$$

However, since the observations are clustered within groups, the residual errors (*ε_ij_*) will not be independent, thus the standard errors and subsequent inference from the linear regression model will not be valid. To address this, a sandwich estimator can be used to estimate cluster-robust variances:

$$V={\left(X^{\prime }X\right)}^{-1}\;\sum_{j=1}^{m}{w^{\prime }}_{j}*w_{j}{\left(X^{\prime }X\right)}^{-1},$$

where *m* is the total number of clusters and $w_{j}=\sum_{k=1}^{n_{j}}e_{k}*x_{k}$ with *x_k_* the row vector of predictors including the intercept and *e_k_* the residual from the linear regression model.^9^

We have developed an R package based on Rcpp^13^ to fit cluster-robust standard errors across CpG sites (https://github.com/jrs95/lmrse).

### Simulation study

We assessed the performance of these approaches for identifying CpGs that change over time in relation to a binary exposure through a simulation study. Specifically, we assessed the following approaches: EWAS at the first time-point only, EWAS at each time-point (considering two strategies for identifying CpGs as being associated: CpGs that are associated with the exposure at any, or at all, time-points), multilevel models with a random intercept, multilevel models with a random intercept and slope, and linear regression with cluster-robust standard errors. This simulation study was performed based on data from the Tsaprouni *et al.* study,^14^ which investigated the relationship between smoking and DNA methylation (data accessible at NCBI GEO database,^15^ accession GSE50660).

In each simulation, 100 CpGs were selected at random, of which six CpGs were simulated to be associated with the binary exposure (Supplementary Figure 1, available as Supplementary Data at *IJE* online). These effects reflect the likely epigenetic associations over time: (i) a constant effect of the exposure but no effect of age on methylation; (ii) a diverging effect of the exposure over time starting at the same baseline value, where, for one level of the exposure, there is no effect of age on methylation; (iii) a diverging effect of the exposure over time starting at the same baseline value; (iv) a constant effect of the exposure as well as an effect of age on methylation; (v) a diverging effect of the exposure over time as well as an effect at baseline; (vi) a converging effect of the exposure over time. The data were simulated using a multilevel model with a random intercept and slope as the underlying data-generating model (Supplementary Material, available as Supplementary Data at *IJE* online).

We considered various numbers of equally spaced repeated measures over childhood and adolescence from the age of 10 to 18 years. The primary analyses were based on five repeated measures, each 2 years apart. In secondary analyses, we also considered three repeats, each 4 years apart, and nine repeats, each 1 year apart.

Statistical power (and Type I error) of the parameters relating to the binary exposure were calculated as the proportion of simulation replicates that have a *p*<1×10^–7^. Type I error was assessed using the 94 CpGs that were not associated with the exposure either at baseline or over time. Relative bias (i.e. $\left(\hat{\beta }-\beta \right)/\beta$) of the parameters related to the exposure was also used to compare the linear regression model with robust standard errors in relation to the multilevel model with both a random intercept and slope. For each simulation scenario, 1000 simulation replicates were performed.

### Application to prenatal exposure to smoking and DNA methylation change

#### Study population

This study used DNA methylation data generated as part of the Avon Longitudinal Study of Parents and Children (ALSPAC).^16^^,^^17^ ALSPAC recruited 14 541 pregnant women with expected delivery dates between April 1991 and December 1992. Of these initial pregnancies, there were 14 062 live births and 13 988 children who were alive at 1 year of age. The study website contains details of all the data that are available through a fully searchable data dictionary (http://www.bris.ac.uk/alspac/researchers/data-access/data-dictionary). Ethical approval for the study was obtained from the ALSPAC Ethics and Law Committee and the Local Research Ethics Committees. As part of the Accessible Resource for Integrated Studies (ARIES) project (http://www.ariesepigenomics.org.uk),^18^ a sub-sample of 1018 ALSPAC child–mother pairs had DNA methylation measured. The ARIES participants were selected based on availability of DNA samples at two time-points for the mother (antenatal and at follow-up when the offspring was in adolescence) and at three time-points for the offspring (neonatal from cord blood, childhood (age 7) and adolescence (age 17)).

#### Laboratory methods, quality control and preprocessing

The laboratory methods and quality-control procedures used have been described elsewhere.^5^ In brief, the DNA methylation wet laboratory and preprocessing analyses were performed at the University of Bristol as part of the ARIES project, where the Infinium HumanMethylation450 BeadChip^8^ was used to measure genome-wide DNA methylation levels at over 485 000 CpG sites. The methylation level at each CpG site was calculated as a beta value: the ratio of the methylated probe intensity and the overall intensity. These beta values range from 0 (no methylation) to 1 (complete methylation). The samples were processed using functional normalization with the meffil package.^19^^,^^20^ Further quality-control procedures are described in the Supplementary Material, available as Supplementary Data at *IJE* online. 

#### Prenatal exposure to smoking

Prenatal exposure to smoking was defined as sustained smoking of the mother during pregnancy. A mother was classified as a sustained smoker if she smoked in the third trimester and at least one of the first two trimesters. The reference group consisted of mothers who had reported not smoking in all three trimesters. We excluded all individuals who smoked in one trimester only (i.e. not sustained), had missing data for more than one trimester or had stopped smoking by the third trimester.

#### Statistical analyses

The cross-sectional and longitudinal approaches were fitted to the three repeated measures of methylation in the offspring (neonatal, at age 7 and at age 17) to investigate the effect of sustained maternal smoking during pregnancy on offspring DNA methylation. An EWAS was fitted at each of the three time-points. Multilevel models (with random intercept and slope) were fitted individually for each CpG, with sustained maternal smoking during pregnancy as the exposure of interest (with a baseline effect and an interaction with age). The linear regression model with robust standard errors takes on the same form as the multilevel models in terms of fixed-effects parameters. All analyses were adjusted for offspring gender, maternal age, pre-pregnancy BMI, pre-pregnancy weight, parity, maternal education, family social class, alcohol intake during pregnancy and paternal smoking, as well as cell counts estimated using the method described by Houseman *et al.*^21^ We further adjusted the models for 20 (time-point specific) surrogate variables to account for residual batch effects.^22^ CpGs were considered to be associated with prenatal exposure to smoking if any parameter related to prenatal smoking was associated at EWAS level of significance (*p* < 1 × 10^–7^). The computational times of performing each strategy were assessed using 100 000 CpGs using 10 cores (2.6 GHz; 4 GB) on a linux server.

All analyses were performed using R (version 3.31).

## Results

### Simulation study

**Figure 1:**
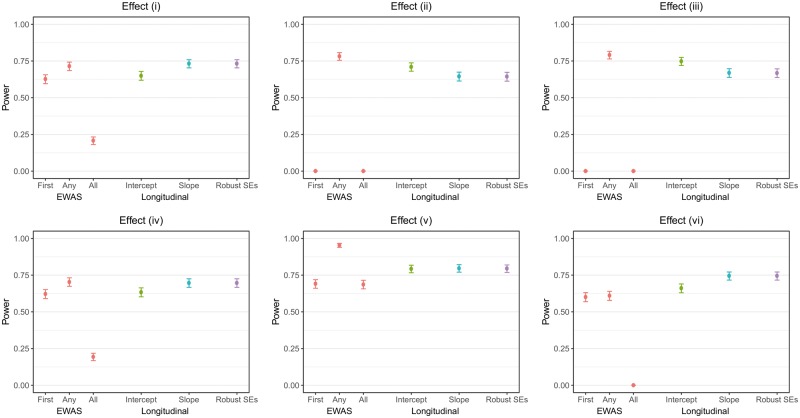
Simulation results for five repeated measures comparing approaches for identifying CpG sites associated with the exposure. Power refers to the proportion of simulation replicates with any parameter related to the exposure with p<1E-7. EWAS, epigenome-wide association study; Intercept, random intercept model; Slope, random intercept and slope model; Robust SEs, linear regression with cluster robust SEs.

Figure 1 displays the simulation results of the statistical power of each strategy (at *p* < 1 × 10^–7^ for any parameter related to the binary exposure in the model) to identify methylated CpGs associated with the binary exposure when there were five repeated measures. The statistical power of each time-specific EWAS and the baseline effect and interaction with age of the exposure in the longitudinal models (at *p*<1×10^–7^) for five repeated measures is shown in Supplementary Figure 2, available as Supplementary Data at *IJE* online. As expected, EWAS at the first time-point failed to identify methylation at CpGs that are not associated with the exposure at baseline but are as time progresses (Figure 1(ii) and (iii)). However, this approach did identify methylated CpGs that are associated with the binary exposure at baseline, so is relevant for identifying CpGs to investigate persistence of an effect over time using multilevel models (Figure 1(i), (iv) and (v)). The approach where only CpGs that are associated with the exposure at all time-points are considered was highly conservative, whereas the approach that selects CpGs that are associated with the exposure at any time-point performed well across the board, and on some occasions outperformed the longitudinal approaches. However, this is likely to come at the expense of a small inflation in Type I error as the number of repeated measures increases.

The simulation results for the multilevel model with only a random intercept differed slightly from the other two longitudinal approaches (Figure 1 and Supplementary Figure 2, available as Supplementary Data at *IJE* online). In particular, there was less power to detect an association at baseline, while there was greater power to detect the interaction between the exposure and age (Supplementary Figure 2, available as Supplementary Data at *IJE* online). This is because the between-individual variability is modelled in the intercept only, making the slope parameter overly precise. This model misspecification manifests itself in inflated Type I error for this model (Supplementary Table 1, available as Supplementary Data at *IJE* online). The multilevel model with random intercept and slope and the linear regression model with robust standard errors yielded very similar results in terms of power, bias and precision (Table 1).

**Table 1:** Simulation results comparing the longitudinal model with random intercept and slope and linear regression models with cluster-robust standard errors for the causal CpGs for five repeated measures

	Longitudinal model with random intercept and slope				Linear regression with cluster-robust SEs			
CpG	*β_01_*		*β_11_*		*β_01_*		*β_11_*	
	Relative bias	SE	Relative bias	SE	Relative bias	SE	Relative bias	SE
(i)	–0.0266 (0.194)	0.0034	NA	0.0008	–0.0269 (0.194)	0.0034	NA	0.0008
	[–0.107,0.076]	(0.0016)		(0.0004)	[–0.105, 0.076]	(0.0016)		(0.0004)
(ii)	NA	0.0033	–0.0330 (0.222)	0.0008	NA	0.0033	–0.0330 (0.222)	0.0008
		(0.0017)	[–0.151, 0.106]	(0.0004)		(0.0017)	[–0.146, 0.106]	(0.0004)
(iii)	NA	0.0033	–0.0200 (0.216)	0.0008	NA	0.0033	–0.0195 (0.217)	0.0008
		(0.0013)	[–0.121, 0.106]	(0.0004)		(0.0014)	[–0.117, 0.107]	(0.0004)
(iv)	–0.0236 (0.195)	0.0033	NA	0.0008	–0.0237 (0.196)	0.0033	NA	0.0008
	[–0.110, 0.083]	(0.0016)		(0.0003)	[–0.111, 0.081]	(0.0016)		(0.0004)
(v)	–0.0294 (0.212)	0.0033	–0.0113 (0.216)	0.0008	–0.0294 (0.212)	0.0033	–0.0112 (0.217)	0.0008
	[–0.112, 0.079]	(0.0014)	[–0.119, 0.108]	(0.0003)	[–0.118, 0.081]	(0.0014)	[–0.123, 0.109]	(0.0003)
(vi)	–0.0258 (0.198)	0.0032	0.0210 (0.302)	0.0007	–0.0257 (0.198)	0.0033	0.0210 (0.304)	0.0007
	[–0.112, 0.084]	(0.0013)	[–0.149, 0.163]	(0.0003)	[–0.108, 0.084]	(0.0013)	[–0.151, 0.167]	(0.0003)

Relative bias refers to the estimated effect minus the underlying effect divided by the underlying effect. Relative bias is given in mean (standard deviation) [lower quantile, upper quantile]. SE is given in mean (standard deviation). The mean bias for the null underlying effects were approximately zero. SE, standard eror; NA, not applicable.

The results for the simulations using three repeated measures and nine repeated measures yielded similar results and inferences to those with five repeated measures (Supplementary Figures 3–6, available as Supplementary Data at *IJE* online). There was increased power of the longitudinal approaches in comparison to cross-sectional approaches as the number of repeated measures increased. However, there were no material differences between the two longitudinal modelling approaches when the number of repeated measures was increased or decreased (Supplementary Figures 3–6 and Supplementary Tables 2 and 3, available as Supplementary Data at *IJE* online).

### Application to prenatal exposure to smoking and DNA methylation change

In ARIES, 724 mother–offspring pairs had information on prenatal exposure to smoking as well as all the other covariates and methylation. Overall, this left 2044 observations in the offspring available for analysis: 645 neonatal from cord blood, 698 during childhood at age 7 and 701 in adolescence at age 17. In the mother–offspring pairs, 650 (89.8%) of the mothers were classified as non-smokers and 74 (10.2%) were classified as sustained smokers during pregnancy (Table 2).

**Table 2:** Differences between individuals in ARIES whose mothers did not smoke in pregnancy compared with sustained smokers

	Smoking status			
Covariate	Non-smoker (*N* = 650)	Sustained smoker (*N* = 74)	*p*-value	Overall (*N* = 724)
Sex				
Male	49.5	47.3	0.81	49.6
Female	50.5	52.7		50.4
Maternal age	30.1 (4.4)	28.2 (5.3)	0.004	29.9 (4.5)
Parity				
0	43.4	48.7	0.66	44.0
1	40.1	35.1		39.6
2+	16.5	16.2		16.4
Maternal education				
CSE or Vocational	11.9	27.0	<0.001	13.4
O-level	33.4	45.9		34.7
A-level	54.7*	27.1*		29.8
Degree	*	*		22.1
Social class				
I or II	67.2	50.0	<0.001	65.5
III (non-manual)	24.0	21.6		23.7
III (manual)	5.7	16.2		6.8
IV or V	3.1	12.2		4.0
Maternal BMI	22.7 (3.7)	22.9 (3.8)	0.70	22.7 (3.7)
Maternal weight	61.5 (10.3)	61.4 (11.0)	0.97	61.5 (10.4)
Alcohol				
Non-drinker	34.0	40.5	0.32	34.7
Drank during pregnancy	66.0	59.5		65.3
Partner smoking				
Non-smoker	82.6	31.1	<0.001	77.3
Smoker	17.4	68.9		22.7

Continuous variables are given in mean (standard deviation) and binary variables are given in %. *Percentage given for ‘A-level’ and ‘Degree’ combined, due to small cell sizes.

Methylation levels at 23 CpGs were associated with prenatal smoking either through time-point specific EWAS or longitudinally (with *p*<1×10^–7^; Table 3), of which 21 have previously been found to be associated with either prenatal or own smoking.^5^^,^^23^^,^^24^ Nineteen CpGs were identified with prenatal exposure to smoking in the longitudinal models either through an association at baseline or with an interaction with age (16 CpGs were identified by the multilevel model, 17 CpGs were identified by linear regression with cluster-robust standard errors, 14 in common). In general, the multilevel models were more precise in estimating the interaction between prenatal smoking and age. However, the effect estimates across all of the CpGs were very similar across both modelling approaches. Four CpGs were solely identified through the longitudinal analyses (cg09662411 and cg14179389 (*GFI1*), cg27462475 (*DOCK9*) and cg04224247 (*WWC3*)). An additional four CpGs were identified through time-point-specific EWAS exclusively (cg02586610 (*SEMA5B*), cg22089736 (*PXT1*), cg19089201 (*MYO1G*) and cg00213123 (*CYP1A1*)); these CpGs were associated with prenatal exposure to smoking at the later time-points (age 7 and age 17). However, two of these CpGs showed little evidence of a longitudinal association (*SEMA5B* and *PXT1*) with all *p*-values > 0.001.

**Table 3:** Differential methylation in blood DNA over childhood and adolescence for the offspring of mothers with sustained smoking in pregnancy compared with non-smokers

			Multilevel model with random intercept and slope						Linear regression with cluster-robust SEs						Associations with individual time-points	Previously found smoking association
			Intercept			Slope			Intercept			Slope				
CpG	Chr:Pos	Gene	β	SE	p	β	SE	p	β	SE	p	β	SE	p		
**Longitudinal approaches**																
cg09662411	1:92946132	*GFI1*	–0.074	0.014	3.0E-07	0.00458	0.00094	1.0E-06	–0.073	0.012	1.6E-09	0.00449	0.00089	4.8E-07	None	Yes
cg18146737	1:92946700	*GFI1*	–0.130	0.020	2.1E-10	0.00789	0.00103	1.7E-14	–0.126	0.020	3.8E-10	0.00778	0.00118	4.4E-11	Cord	Yes
cg12876356	1:92946825	*GFI1*	–0.109	0.020	6.7E-08	0.00544	0.00109	5.5E-07	–0.106	0.017	1.3E-09	0.00525	0.00103	3.9E-07	Cord	Yes
cg09935388	1:92947588	*GFI1*	–0.134	0.016	2.9E-16	0.00717	0.00086	6.9E-17	–0.134	0.017	1.0E-15	0.00703	0.00098	9.7E-13	Cord	Yes
cg14179389	1:92947961	*GFI1*	–0.059	0.011	2.4E-08	0.00110	0.00068	1.0E-01	–0.060	0.010	2.7E-09	0.00106	0.00073	1.4E-01	None	Yes
cg05575921	5:373378	*AHRR*	–0.068	0.006	1.2E-29	0.00280	0.00042	2.2E-11	–0.069	0.007	5.8E-25	0.00283	0.00048	4.1E-09	Cord, Age 17	Yes
cg22132788	7:45002486	*MYO1G*	0.048	0.009	2.9E-08	0.00243	0.00060	5.0E-05	0.043	0.007	1.8E-09	0.00259	0.00067	1.1E-04	Age 7, Age 17	Yes
cg04180046	7:45002736	*MYO1G*	0.093	0.012	2.4E-14	–0.00001	0.00084	9.9E-01	0.094	0.012	2.7E-14	–0.00009	0.00099	9.3E-01	All	Yes
cg12803068	7:45002919	*MYO1G*	0.100	0.014	3.2E-12	0.00107	0.00075	1.5E-01	0.100	0.013	9.8E-15	0.00109	0.00078	1.6E-01	All	Yes
cg25949550	7:145814306	*CNTNAP2*	–0.016	0.003	2.6E-07	0.00005	0.00024	8.4E-01	–0.015	0.003	4.4E-08	0.00004	0.00021	8.5E-01	Cord, Age 17	Yes
cg27462475	13:99736304	*DOCK9*	0.013	0.004	2.9E-03	–0.00122	0.00034	3.4E-04	0.013	0.003	1.4E-04	–0.00125	0.00022	7.5E-09	None	Yes
cg05549655	15:75019143	*CYP1A1*	0.043	0.005	2.7E-15	–0.00009	0.00035	8.0E-01	0.043	0.006	4.9E-14	–0.00008	0.00036	8.1E-01	All	Yes
cg17852385	15:75019188	*CYP1A1*	0.046	0.008	7.2E-08	0.00048	0.00059	4.2E-01	0.047	0.010	1.3E-06	0.00041	0.00062	5.1E-01	Age 7, Age 17	Yes
cg13570656	15:75019196	*CYP1A1*	0.067	0.012	3.8E-08	0.00018	0.00078	8.2E-01	0.068	0.014	1.7E-06	0.00007	0.00077	9.2E-01	Age 7	Yes
cg12101586	15:75019203	*CYP1A1*	0.081	0.011	1.2E-12	–0.00105	0.00075	1.6E-01	0.084	0.011	1.1E-13	–0.00124	0.00065	5.7E-02	Cord	Yes
cg22549041	15:75019251	*CYP1A1*	0.101	0.015	1.1E-11	–0.00141	0.00106	1.8E-01	0.105	0.017	2.6E-10	–0.00156	0.00118	1.9E-01	Cord, Age 7	Yes
cg11924019	15:75019283	*CYP1A1*	0.047	0.007	8.1E-11	–0.00013	0.00043	7.6E-01	0.048	0.008	4.8E-10	–0.00022	0.00043	6.1E-01	All	Yes
cg18092474	15:75019302	*CYP1A1*	0.094	0.016	8.2E-09	0.00000	0.00115	1.0E+00	0.093	0.015	4.2E-10	0.00013	0.00119	9.2E-01	Cord	Yes
cg04224247	X:9984515	*WWC3*	–0.050	0.009	6.0E-09	0.00085	0.00057	1.4E-01	–0.050	0.009	9.9E-09	0.00085	0.00060	1.6E-01	None	Yes
**Cross-sectional approaches**																
cg02586610	3:122745092	*SEMA5B*	–0.002	0.002	3.7E-01	0.00064	0.00020	1.3E-03	–0.002	0.002	2.7E-01	0.00064	0.00026	1.5E-02	Age 17	No
cg22089736	6:36359367	*PXT1*	–0.005	0.003	1.0E-01	–0.00044	0.00023	5.7E-02	–0.004	0.003	2.8E-01	–0.00051	0.00024	2.9E-02	Age 7	No
cg19089201	7:45002287	*MYO1G*	0.039	0.009	2.8E-05	0.00289	0.00073	7.2E-05	0.036	0.009	5.5E-05	0.00300	0.00066	6.3E-06	Age 17	Yes
cg00213123	15:75019070	*CYP1A1*	0.021	0.005	6.1E-05	0.00012	0.00038	7.5E-01	0.021	0.005	2.4E-05	0.00012	0.00038	7.6E-01	Age 7	Yes

Time-points: cord refers to methylation levels in cord blood; Age 7 refers to methylation levels in the offspring at the 7-year clinic; Age 17 refers to methylation levels in the offspring at the 17-year clinic. Previously found smoking association refers to associations found in Richmond *et al.*,^5^ Joubert *et al.*^24^ and Joehanes *et al.*^23^ None of the CpG sites identified is in the list of false or SNP probes in the Chen *et al.* paper.^30^ Chr:Pos, build 37 chromosome position; SE, standard error.

**Figure 2:**
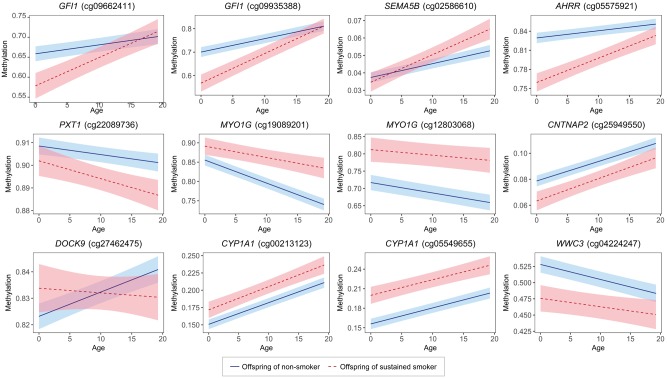
Longitudinal trajectories of methylation for a subset of the CpGs associated with prenatal smoking during pregnancy (Table 3; Figures S7 & S8) in the offspring of non-smokers and sustained smokers during pregnancy from birth to age 20. The solid and dashed lines are the longitudinal models for offspring of nonsmokers and sustained smokers respectively (the bands represent the 95% confidence intervals).

The longitudinal associations for a key subset of the 23 methylated CpGs that are associated with prenatal smoking are displayed in Figure 2 (all 23 are presented in Supplementary Figures 7 and 8, available as Supplementary Data at *IJE* online). Some of the methylated CpGs that are associated with prenatal exposure to smoking at baseline resolve over childhood and adolescence to a similar methylation level (e.g. *GFI1*, *AHRR* and *WWC3*). Other associations remained reasonably constant over time (e.g. *CNTNAP2*, *MYO1G* and *CYP1A1*).

The computational time required to complete each approach for 100 000 CpGs were as follows: 15 seconds for EWAS at baseline only, 45 seconds for EWAS at each time-point, 1894 minutes for the multilevel model with a random intercept and slope, and 26 minutes for the linear regression model with cluster-robust standard errors.

## Discussion

In this study, we have investigated approaches for identifying epigenetic change between DNA methylation and an exposure. These approaches were tested in simulations and were used to investigate the effect of sustained maternal smoking during pregnancy on offspring DNA methylation change during childhood and adolescence.

Out of the three approaches that involved performing an EWAS at baseline or at each time-point, the approach of taking forward CpGs that are associated at any time-point performed best. This approach will have increased Type I error as the number of repeats increase (unless appropriately accounted for); however, as the Bonferroni significance threshold used in EWAS is already conservative, this is unlikely to be problem in practice. The multilevel model with only a random intercept had increased Type I error, through inflated power to detect a difference in slope between those who are exposed and not exposed. Thus, this model is likely to be an inappropriate choice of model to fit across all CpGs. Linear regression with cluster-robust standard errors performed well in comparison to the multilevel model with a random intercept and slope. This approach was also much more computationally efficient (74 times faster) than multilevel models. Further advantages of this approach are consistent convergence and no dependence on choice of random-effects parameters.^11^

Well-known associations of prenatal smoking were identified through EWAS and through longitudinal analyses (*GFI1*, *AHRR*, *MYO1G* and *CYP1A1*),^5^^,^^23^^,^^24^ as well as a few potentially novel associations (*SEMA5B* and *PXT1*). Three-fifths of the associations that were identified using the cross-sectional approaches and the longitudinal approaches overlap (15 out of 23), with the cross-sectional approaches identifying an additional four CpGs (of which two showed little evidence of a longitudinal association in follow-up analyses or an association in the literature and therefore might be false positives) and the longitudinal approaches identifying a further four CpGs. Some of these longitudinal associations resolved over time (e.g. *GFI1*, *AHRR* and *WWC3*), while others remained constant (e.g. *CNTNAP2*, *MYO1G* and *CYP1A1*).

This study is also applicable to other areas of medical research where repeated measures of high-dimensional phenotypes are available, such as metabolomics.^25^ Indeed, the results of this study are broadly generalizable to any study where large numbers of longitudinal analyses need to be performed, including genome-wide association studies (GWAS) with repeated measures of an outcome.^26^^,^^27^ However, fast approximate (two-stage) methods are available for GWAS of a longitudinal outcome where: (i) a single longitudinal model of the outcome is fitted with time/age and covariates only and (ii) the subject-specific beta estimates of time/age from this model are then tested against the genetic variants using linear regression.^28^^,^^29^

The limitations of this study also warrant consideration. In the simulations and applied example, only a binary exposure was considered, although we fully expect these results to extrapolate to continuous exposures. The application of the approaches to prenatal exposure to smoking also has several limitations, especially with regard to residual confounding. In particular, the CpGs where the association with prenatal exposure to smoking diverged over time are perhaps more likely to be due to other factors (e.g. exposure to smoking during childhood and adolescence), which are not captured fully in the questionnaire data available. The ARIES cohort is also not selected at random from the full ALSPAC cohort and, as such, the results from this study may not generalizable to the full ALSPAC cohort or the general population.

In summary, linear regression with cluster-robust standard errors is a computationally efficient alternative to multilevel models, yielding similar effect estimates and overall inference, although performing an EWAS at each time-point to identify CpGs is also a practical alternative to fitting multilevel models across the epigenome.

## Supplementary Data

Supplementary data are available at *IJE* online.

## Funding

This work was supported by an Medical Research Council Methodology Research Grant (grant number MR/M025020/1). Work was performed in the Medical Research Council Integrative Epidemiology Unit (grant numbers MC_UU_12013/2, MC_UU_12013/8 and MC_UU_12013/9).

## Supplementary Material

Supplementary Data
