# Supplementary material for: Longitudinal analysis strategies for modelling epigenetic trajectories
Source: Int J Epidemiol. 2018 Feb 16;47(2):516–25. doi: 10.1093/ije/dyy012 (PMC5913606; doi:10.1093/ije/dyy012)

Longitudinal analysis strategies for modelling epigenetic trajectories: supplementary material

James R Staley^1*^, Matthew Suderman^1^, Andrew J Simpkin^1^, Tom R Gaunt^1^, Jon Heron^1^, Caroline L Relton^1^, and Kate Tilling^1^.

^1^MRC Integrative Epidemiology Unit, Bristol Medical School, University of Bristol, Bristol, BS8 2BN, UK.

Short title: Longitudinal modelling of DNA methylation

Conflicts of interest: None

Correspondence:

Dr James R Staley

MRC Integrative Epidemiology Unit, Bristol Medical School, University of Bristol, Bristol, BS8 2BN, UK.

Email: [js16174@bristol.ac.uk](mailto:js16174@bristol.ac.uk)

Telephone: +44 (0)117 331 0098

Fax: +44 (0)117 331 4052

# Supplementary Text

## Epigenome-wide association studies

Epigenome-wide association studies (EWAS) have been used to assess associations of DNA methylation at CpG sites from across the genome with diseases and traits (1,2). DNA methylation is usually quantified between 0 and 1, and represents the proportion of methylated DNA molecules at the CpG site in the measured tissue. The initial analyses involve univariate testing of each CpG site (e.g. on the Illumina HumanMethylation 450 array there are 485,000 CpG sites (3)) to identify DNA methylation that is associated with an exposure and/or a phenotype (4) while accounting for multiple testing (a Bonferroni corrected *p*-value threshold of $\sim1\times{10}^{-7}$ is often used for studies based on the Illumina HumanMethylation 450 array). Methylation at CpG sites is often treated as the outcome (i.e. a possible consequence of the trait) (4), where mean levels of methylation are regressed against the exposure using a linear regression model. These analyses are usually adjusted for batch effects and other technical covariates (5), as well as for cell composition (6) and other potential confounding factors such as age, gender and other characteristics. Further downstream analyses including differential methylated region analyses where adjacent CpG sites are tested together (7) and functional and gene set enrichment analyses (4) are also often performed.

## Simulation model

The simulation model was as follows,

$$y_{ij}^{(k)}= \left( \beta_{00}^{(k)}+ \beta_{01}^{(k)}z_{j}+u_{0j}^{(k)} \right)+ \left( \beta_{10}^{(k)}+ \beta_{11}^{(k)}z_{j}+u_{1j}^{(k)} \right)x_{ij}+\epsilon_{ij}^{(k)} ,$$

where $y_{ij}^{(k)}$ are the repeated measures of methylation for the $k$-th CpG site of the $i$-th repeated measures for the $j$-th individual; $x_{ij}$ is the age of the individual when methylation was measured ($x_{1j}\sim U(10,10.99)$ and $x_{ij}=x_{1j}$ + number of years between measures of methylation); and $z_{j}$ is the binary exposure ($z_{j} \sim\mathrm{Bernoulli}(0.5)$). $\beta_{00}^{(k)}$, $\beta_{01}^{(k)}$, $\beta_{10}^{(k)}$ and $\beta_{11}^{(k)}$ are the underlying effects; $\beta_{00}^{(k)}$ was set as the mean methylation level for the $k$-th random CpG site based on the Tsaprouni *et al.* dataset (8) and $\beta_{01}^{(k)}$, $\beta_{10}^{(k)}$ and $\beta_{11}^{(k)}$ were based on the underlying simulation model. To ensure that the methylation beta-value was in the interval [0,1], we chose $\beta_{01}^{(k)}$, $\beta_{10}^{(k)}$ and $\beta_{11}^{(k)}$ (unless equal to 0 in the underlying model) as a proportion of the variability of the CpG ($\sigma_{00}^{\left( k \right)}$) according to $\beta_{00}^{(k)}$. In particular, for underlying effects (i) to (v),

$\beta_{01}^{(k)}=\left\{ \begin{matrix} 1.05\times\sigma_{00}^{\left( k \right)} & \mathrm{if}\min\left( \beta_{00}^{\left( k \right)},1-\beta_{00}^{\left( k \right)} \right)=\beta_{00}^{(k)} \\ -1.05\times\sigma_{00}^{\left( k \right)} & \mathrm{if}\min\left( \beta_{00}^{\left( k \right)}, 1-\beta_{00}^{\left( k \right)} \right)\neq\beta_{00}^{(k)} \end{matrix} \right.$ , $\beta_{10}^{(k)}= \left\{ \begin{matrix} 0.2\times\sigma_{00}^{\left( k \right)} & \mathrm{if}\min\left( \beta_{00}^{\left( k \right)},1-\beta_{00}^{\left( k \right)} \right)=\beta_{00}^{(k)} \\ -0.2\times\sigma_{00}^{\left( k \right)} & \mathrm{if}\min\left( \beta_{00}^{\left( k \right)}, 1-\beta_{00}^{\left( k \right)} \right)\neq\beta_{00}^{(k)} \end{matrix} \right.$ , $\beta_{11}^{(k)}= \left\{ \begin{matrix} 0.2\times\sigma_{00}^{\left( k \right)} & \mathrm{if}\min\left( \beta_{00}^{\left( k \right)},1-\beta_{00}^{\left( k \right)} \right)=\beta_{00}^{(k)} \\ -0.2\times\sigma_{00}^{\left( k \right)} & \mathrm{if}\min\left( \beta_{00}^{\left( k \right)}, 1-\beta_{00}^{\left( k \right)} \right)\neq\beta_{00}^{(k)} \end{matrix} \right.$ ,

and for underlying effect (vi) where $\beta_{01}^{(k)}$ is the same as in (2), $\beta_{10}^{(k)}=0.065\times\sigma_{00}^{\left( k \right)}$ and $\beta_{11}^{(k)}=-2\times\beta_{10}^{(k)}$.

The $u$’s were generated using a multivariate normal distribution using the variance-covariance matrix ($\Omega$) from the Tsaprouni *et al.* dataset (the variance component for the $u_{0j}$’s was set as $\Omega$, the variance component for the $u_{1j}$’s was set as $0.1\times\Omega$, and the $u_{0j}$’s and $u_{1j}$’s were assumed to be uncorrelated). The individual-level error component was also normally distributed, $\epsilon_{ij}\sim N(0, {0.05}^{2})$.

## Quality control and pre-processing procedures in ARIES

Cord blood and peripheral blood samples (whole blood, buffy coats or blood spots) were collected according to standard procedures. The DNA methylation wet laboratory and pre-processing analyses were performed at the University of Bristol as part of the ARIES project. Following extraction, DNA was bisulphiteconverted using the Zymo EZ DNA MethylationTM kit (Zymo, Irvine, CA, USA). Following conversion, genome-wide methylation of over 485,000 CpG sites were measured using the Infinium HumanMethylation450 BeadChip according to the standard protocol. The arrays were scanned using an Illumina iScan and initial quality review was assessed using GenomeStudio (version 2011.1).

Samples from all time points in ARIES were distributed across slides using a semi-random approach (sampling criteria were in place to ensure that all time points were represented on each array) to minimize the possibility of confounding by batch effects. In addition, during the data generation process a wide range of batch variables were recorded in a purpose-built laboratory information management system (LIMS). The main batch variable was found to be the bisulphite conversion (BCD) plate number. Samples were converted in batches of 48 samples and each batch identified by a plate number. The LIMS also reported quality control (QC) metrics from the standard control probes on the 450K BeadChip for each sample. Samples with more than 5% of probes that have detection $p>$ 0.01 were excluded from the analysis. As an additional QC step genotype probes were compared with SNP-chip data from the same individual to identify and remove any sample mismatches. For individuals with no genome-wide SNP data, samples were flagged if there was a sex-mismatch based on X and Y chromosome methylation.

In addition to these QC steps, probes that had detection $p>$ 0.01 for more than 5% of samples were excluded from analysis. After excluding these probes, a total of 482,855 CpG sites were included in the main analysis. Raw probe intensities were normalized using functional normalization with the meffil package (9,10).

## References

1. Rakyan VK, Down TA, Balding DJ, Beck S. Epigenome-wide association studies for common human diseases. Nat Rev Genet. 2011; 12:529-41.

2. Flanagan JM. Epigenome-wide association studies (EWAS): past, present, and future. Methods Mol Biol. 2015; 1238:51-63.

3. Dedeurwaerder S, Defrance M, Calonne E, Denis H, Sotiriou C, Fuks F. Evaluation of the Infinium Methylation 450K technology. Epigenomics. 2011; 3:771-84.

4. Michels KB, Binder AM, Dedeurwaerder S*, et al.* Recommendations for the design and analysis of epigenome-wide association studies. Nat Methods. 2013; 10:949-55.

5. Johnson WE, Li C, Rabinovic A. Adjusting batch effects in microarray expression data using empirical Bayes methods. Biostatistics. 2007; 8:118-27.

6. Houseman EA, Molitor J, Marsit CJ. Reference-free cell mixture adjustments in analysis of DNA methylation data. Bioinformatics. 2014; 30:1431-9.

7. Jaffe AE, Murakami P, Lee H*, et al.* Bump hunting to identify differentially methylated regions in epigenetic epidemiology studies. Int J Epidemiol. 2012; 41:200-9.

8. Tsaprouni LG, Yang TP, Bell J*, et al.* Cigarette smoking reduces DNA methylation levels at multiple genomic loci but the effect is partially reversible upon cessation. Epigenetics. 2014; 9:1382-96.

9. Fortin JP, Labbe A, Lemire M*, et al.* Functional normalization of 450k methylation array data improves replication in large cancer studies. Genome Biol. 2014; 15:503.

10. Min J, Hemani G, Davey Smith G, Relton CL, Suderman M. Meffil: efficient normalisation and analysis of very large DNA methylation samples. bioRxiv. 2017.

# Supplementary Tables and Figures

Table S1: Type I error rates for the modelling strategies across the 94,000 CpGs with no effect.

| Repeats | EWAS | | | Longitudinal models | | Linear regression with cluster robust SEs |
| --- | --- | --- | --- | --- | --- | --- |
|  | First | Any | All | Intercept | Slope |  |
| 3 | 0 | 0 | 0 | 0.00001 | 0 | 0 |
| 5 | 0 | 0 | 0 | 0.00018 | 0 | 0 |
| 9 | 0 | 0 | 0 | 0.00267 | 0 | 0 |
| Type I error rate was calculated as the proportion of simulation replicates with any parameter related to the exposure with $p<1\times{10}^{-7}$. Repeats refers to the number of measurements for each individual. EWAS, epigenome-wide association study; Intercept, random intercept model; Slope, random intercept and slope model. | | | | | | |

Table S2: Simulation results comparing the longitudinal model with random intercept and slope and linear regression models with cluster robust standard errors for the causal CpGs for three repeated measures.

| CpG | Longitudinal model with random intercept and slope | | | | Linear regression with cluster robust SEs | | | |
| --- | --- | --- | --- | --- | --- | --- | --- | --- |
|  | $\beta_{01}$ | | $\beta_{11}$ | | $\beta_{01}$ | | $\beta_{11}$ | |
|  | Relative Bias | SE | Relative Bias | SE | Relative Bias | SE | Relative Bias | SE |
| (i) | -0.0317 (0.213) [-0.127, 0.076] | 0.0036 (0.0012) | NA | 0.0008 (0.0003) | -0.0320 (0.213) [-0.126, 0.074] | 0.0036 (0.0012) | NA | 0.0008 (0.0003) |
| (ii) | NA | 0.0036 (0.0013) | -0.0131 (0.222) [-0.121, 0.120] | 0.0008 (0.0003) | NA | 0.0036 (0.0014) | -0.0130 (0.223) [-0.122, 0.121] | 0.0008 (0.0003) |
| (iii) | NA | 0.0036 (0.0012) | -0.0107 (0.227) [-0.129, 0.118] | 0.0008 (0.0003) | NA | 0.0036 (0.0012) | -0.0115 (0.228) [-0.134, 0.116] | 0.0008 (0.0003) |
| (iv) | -0.0282 (0.226) [-0.113, 0.085] | 0.0037 (0.0013) | NA | 0.0008 (0.0004) | -0.0282 (0.226) [-0.115, 0.083] | 0.0037 (0.0013) | NA | 0.0008 (0.0004) |
| (v) | -0.0305 (0.213) [-0.118, 0.086] | 0.0037 (0.0015) | -0.0109 (0.227) [-0.131, 0.109] | 0.0008 (0.0003) | -0.0303 (0.213) [-0.118, 0.0857] | 0.0037 (0.0015) | -0.0113 (0.229) [-0.131, 0.107] | 0.0008 (0.0004) |
| (vi) | -0.0241 (0.213) [-0.104, 0.089] | 0.0037 (0.0015) | 0.0291 (0.304) [-0.154, 0.179] | 0.0008 (0.0004) | -0.0241 (0.213) [-0.104, 0.089] | 0.0037 (0.0015) | 0.0292 (0.304) [-0.159, 0.181] | 0.0008 (0.0004) |
| Relative bias refers to the estimated effect minus the underlying effect divided by the underlying effect. Relative bias is given in mean (standard deviation) [lower quantile, upper quantile]. SE is given in mean (standard deviation). The mean bias for the null underlying effects were approximately zero. SE, standard erors; NA, not applicable. | | | | | | | | |

Table S3: Simulation results comparing the longitudinal model with random intercept and slope and linear regression models with cluster robust standard errors for the causal CpGs for nine repeated measures.

| CpG | Longitudinal model with random intercept and slope | | | | Linear regression with cluster robust SEs | | | |
| --- | --- | --- | --- | --- | --- | --- | --- | --- |
|  | $\beta_{01}$ | | $\beta_{11}$ | | $\beta_{01}$ | | $\beta_{11}$ | |
|  | Relative Bias | SE | Relative Bias | SE | Relative Bias | SE | Relative Bias | SE |
| (i) | -0.0256 (0.176) [-0.096, 0.066] | 0.0029 (0.0015) | NA | 0.0007 (0.0004) | -0.0259 (0.177) [-0.098, 0.068] | 0.0029 (0.0015) | NA | 0.0007 (0.0004) |
| (ii) | NA | 0.0028 (0.0014) | -0.0206 (0.196) [-0.130, 0.104] | 0.0007 (0.0004) | NA | 0.0028 (0.0014) | -0.0195 (0.197) [-0.127, 0.101] | 0.0007 (0.0004) |
| (iii) | NA | 0.0029 (0.0016) | -0.0284 (0.193) [-0.125, 0.090] | 0.0007 (0.0004) | NA | 0.0029 (0.0017) | -0.0293 (0.194) [-0.130, 0.092] | 0.0007 (0.0004) |
| (iv) | -0.0275 (0.166) [-0.090, 0.062] | 0.0029 (0.0012) | NA | 0.0007 (0.0004) | -0.0267 (0.166) [-0.090, 0.061] | 0.0029 (0.0013) | NA | 0.0007 (0.0004) |
| (v) | -0.0246 (0.191) [-0.097, 0.069] | 0.0029 (0.0015) | -0.0061 (0.194) [-0.113, 0.098] | 0.0007 (0.0004) | -0.0246 (0.191) [-0.096, 0.069] | 0.0030 (0.0016) | -0.0061 (0.194) [-0.115, 0.099] | 0.0007 (0.0004) |
| (vi) | -0.0173 (0.164) [-0.089, 0.067] | 0.0029 (0.0014) | 0.0165 (0.266) [-0.136, 0.162] | 0.0007 (0.0004) | -0.0175 (0.164) [-0.091, 0.068] | 0.0029 (0.0014) | 0.0169 (0.268) [-0.140, 0.164] | 0.0007 (0.0004) |
| Relative bias refers to the estimated effect minus the underlying effect divided by the underlying effect. Relative bias is given in mean (standard deviation) [lower quantile, upper quantile]. SE is given in mean (standard deviation). The mean bias for the null underlying effects were approximately zero. SE, standard erors; NA, not applicable. | | | | | | | | |

Figure S1: Simulation effects for the 6 causal CpG sites. The black lines represent the fit of the estimated longitudinal model with random intercept and slope for z equal to 0 and 1 and the black points are the EWAS at each clinic for z equal to 0 and 1.


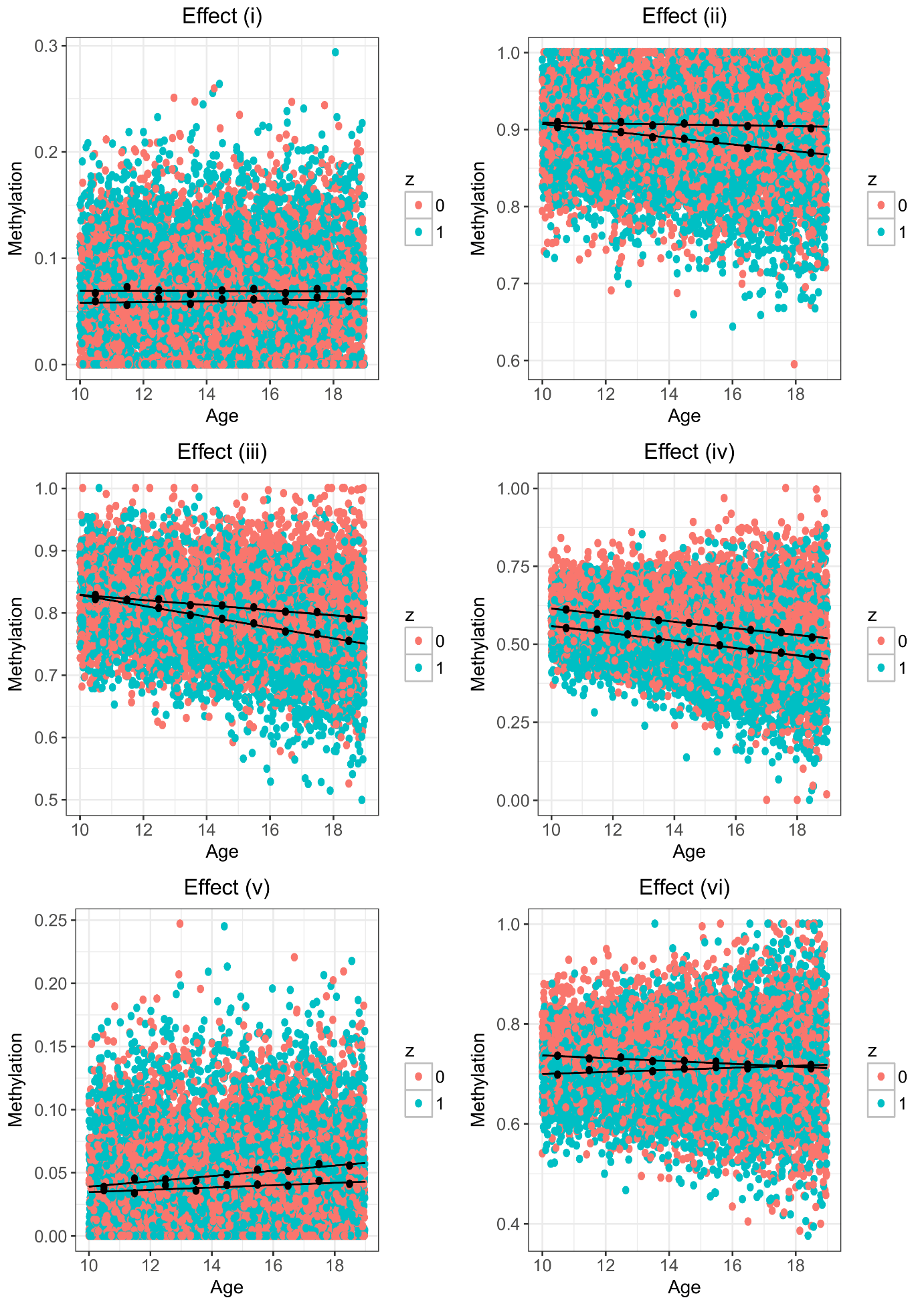


Figure S2: Simulation results for five repeated measures comparing EWAS at each time point and the longitudinal approaches. Power refers to the proportion of simulation replicates with $p<1\times{10}^{-7}$. Intercept, random intercept model; Slope, random intercept and slope model; Robust SEs, linear regression with cluster robust SEs. Z is the baseline effect of the exposure and Z×A is the interaction between the exposure and age.


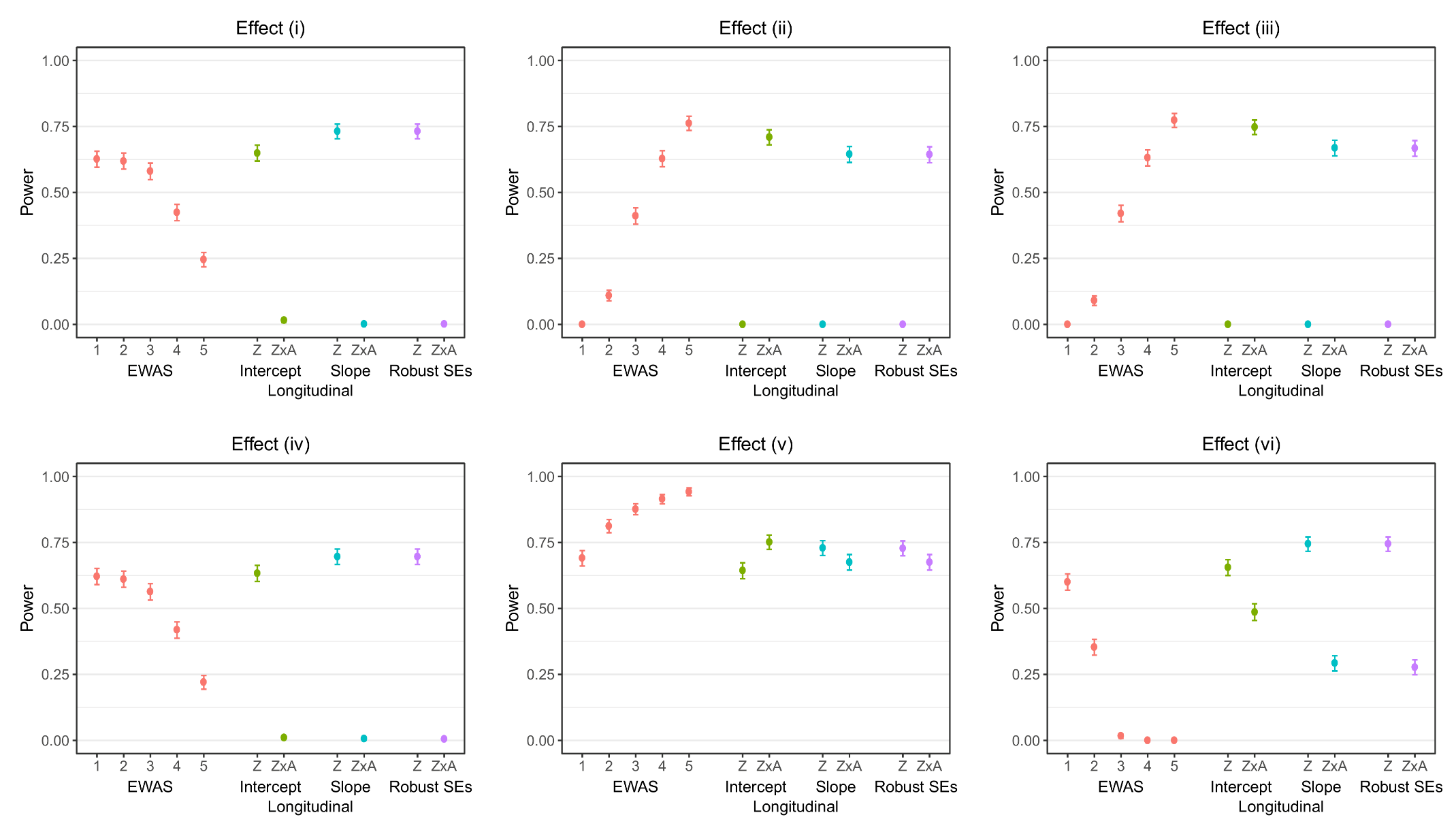


Figure S3: Simulation results for three repeated measures comparing approaches for identifying CpG sites associated with the exposure. Power refers to the proportion of simulation replicates with any parameter related to the exposure with $p<1\times{10}^{-7}$. EWAS, epigenome-wide association study; Intercept, random intercept model; Slope, random intercept and slope model; Robust SEs, linear regression with cluster robust SEs.


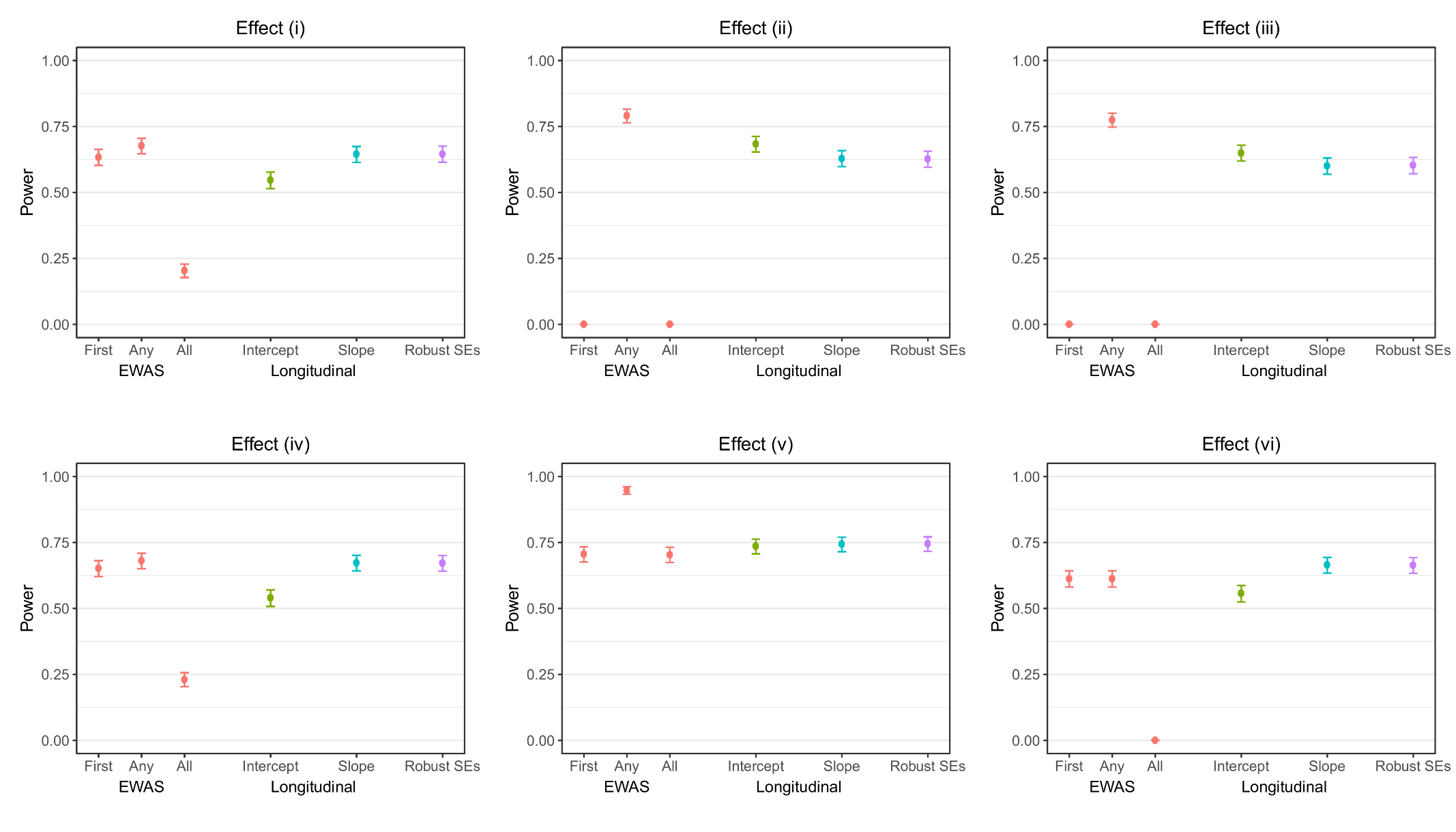


Figure S4: Simulation results for three repeated measures comparing EWAS at each time point and the longitudinal approaches. Power refers to the proportion of simulation replicates with $p<1\times{10}^{-7}$. Intercept, random intercept model; Slope, random intercept and slope model; Robust SEs, linear regression with cluster robust SEs. Z is the baseline effect of the exposure and Z×A is the interaction between the exposure and age.


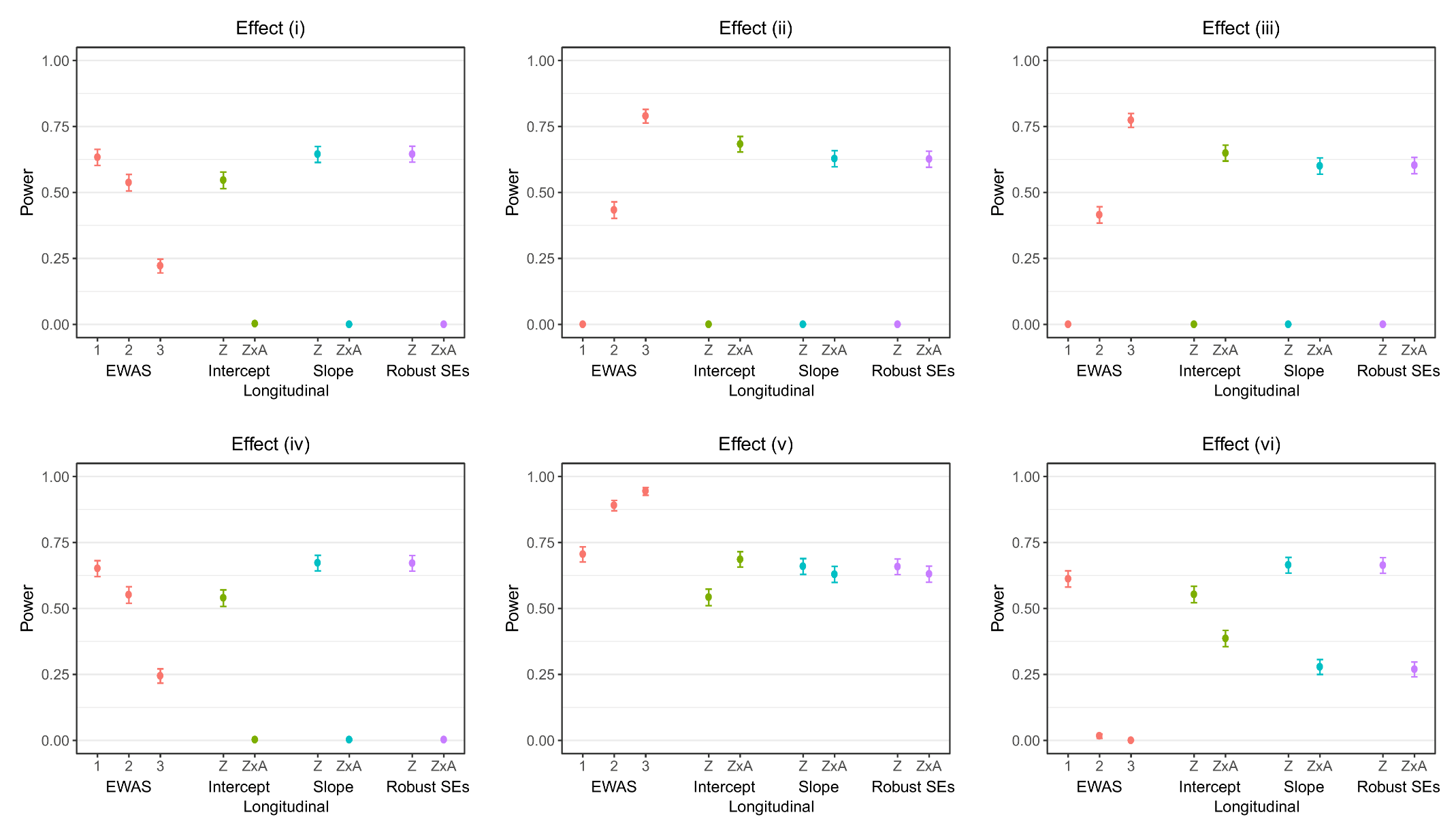


Figure S5: Simulation results for nine repeated measures comparing approaches for identifying CpG sites associated with the exposure. Power refers to the proportion of simulation replicates with any parameter related to the exposure with $p<1\times{10}^{-7}$. EWAS, epigenome-wide association study; Intercept, random intercept model; Slope, random intercept and slope model; Robust SEs, linear regression with cluster robust SEs.


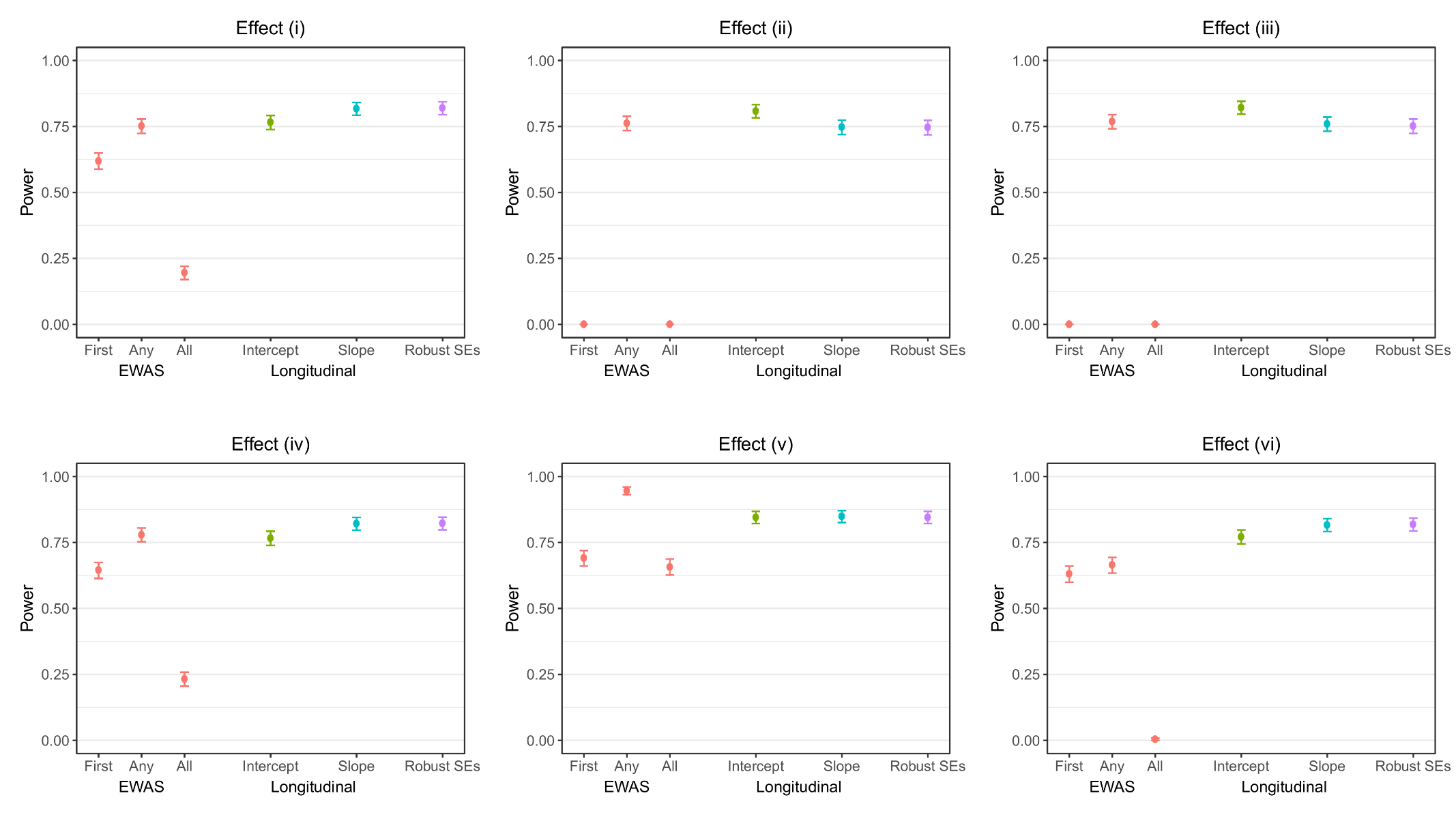


Figure S6: Simulation results for nine repeated measures comparing EWAS at each time point to longitudinal models and linear regression with cluster robust standard errors. Power refers to the proportion of simulation replicates with $p<1\times{10}^{-7}$. EWAS, epigenome-wide association study; Intercept, random intercept model; Slope, random intercept and slope model; Robust SEs, linear regression with cluster robust SEs.


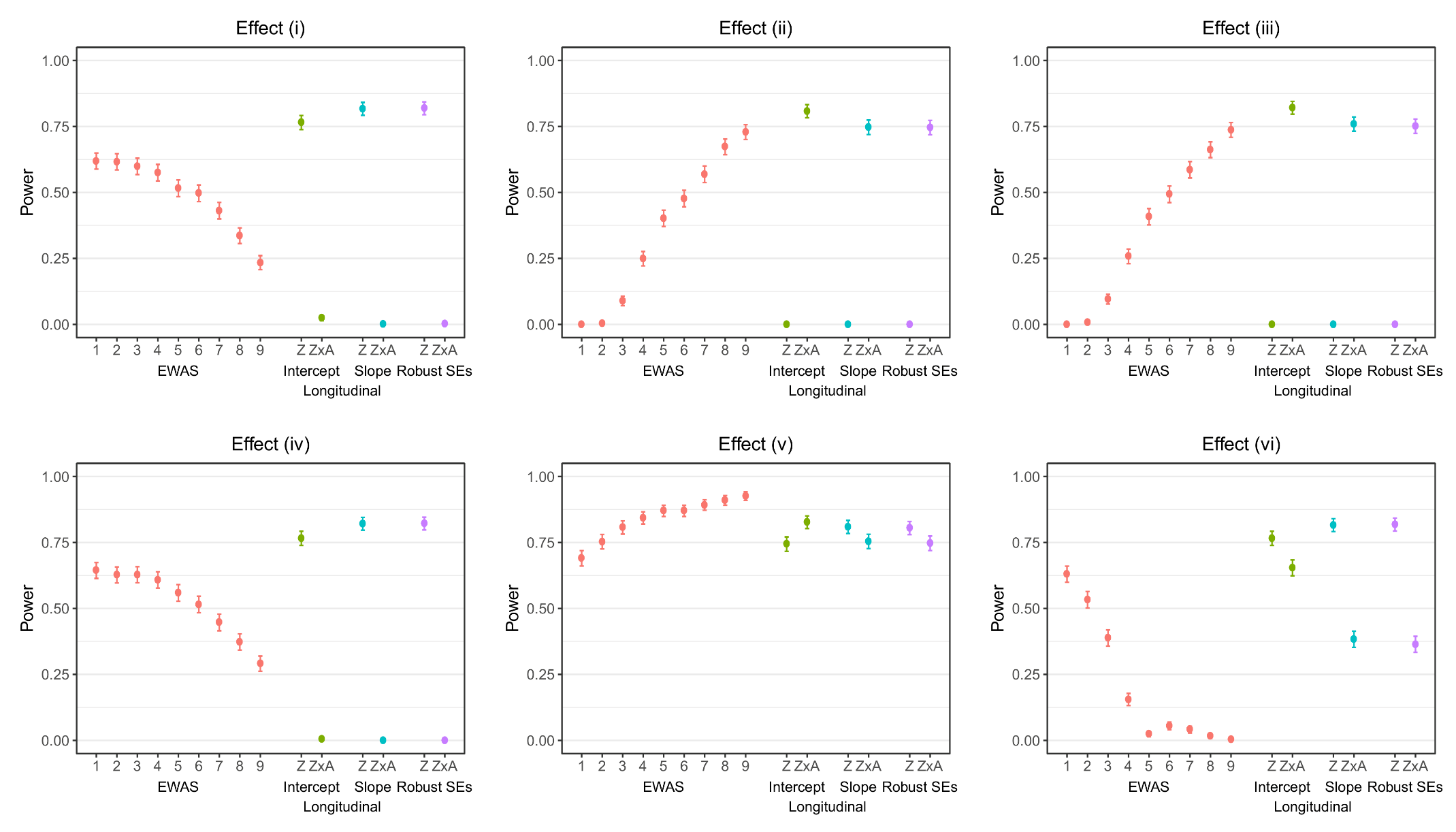


Figure S7: Longitudinal trajectories of methylation for the first 12 CpGs (based on genome position) associated with prenatal smoking during pregnancy (Table 3) in the offspring of non-smokers and sustained smokers during pregnancy from birth to age 20. The blue and red lines are the longitudinal models for offspring of non-smokers and sustained smokers respectively (the bands represent the 95% confidence intervals).


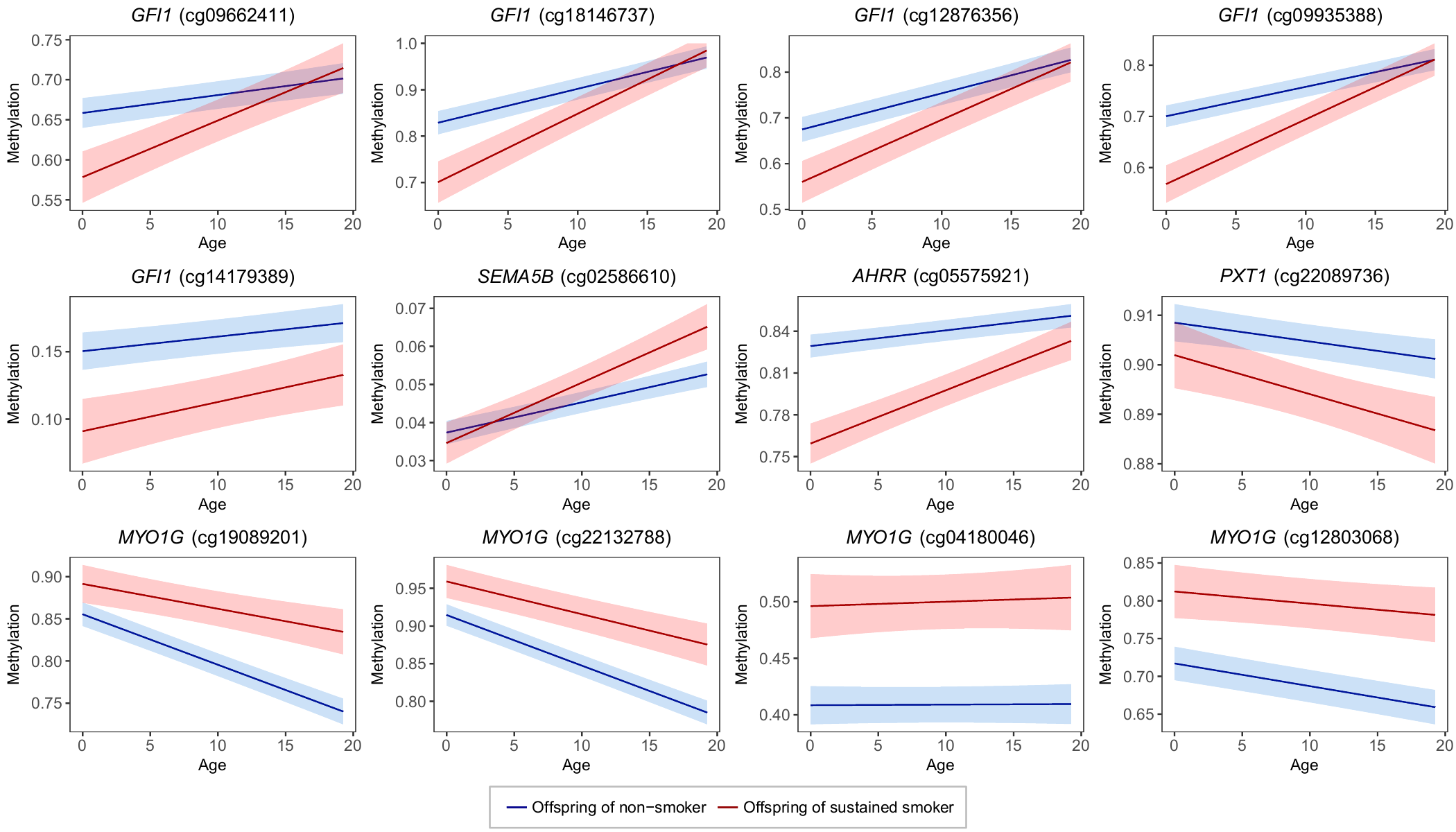


Figure S8: Longitudinal trajectories of methylation for the last 11 CpGs (based on genome position) associated with prenatal smoking during pregnancy (Table 3) in the offspring of non-smokers and sustained smokers during pregnancy from birth to age 20. The blue and red lines are the longitudinal models for offspring of non-smokers and sustained smokers respectively (the bands represent the 95% confidence intervals).


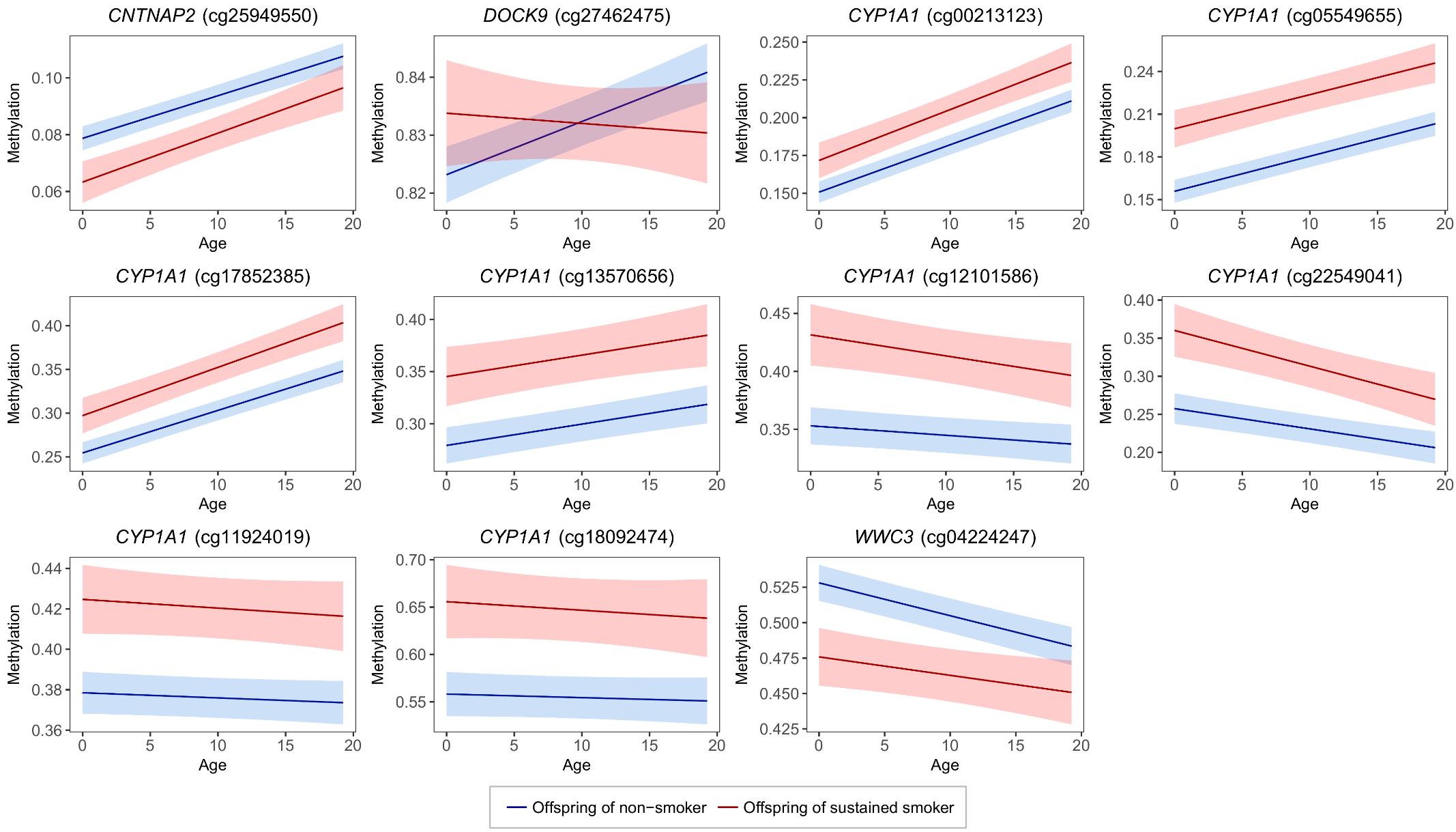

Supplement: Supplementary Data [file dyy012_ije-2017-08-0932-file007.docx]
